# Plant polyphenols as context-dependent modulators of cellular signalling networks

**DOI:** 10.3389/fnut.2026.1885983

**Published:** 2026-08-21

**Authors:** Zhenzhen Hou, Zimeng Cheng, Bin Xue

**Affiliations:** 1 School of Food & Pharmaceutical Science and Technology, Guangzhou College of Technology and Business, Guangzhou, China; 2 College of Food Science, South China Agricultural University, Guangzhou, Guangdong, China

**Keywords:** AMPK, bioavailability, MAPK/ERK, NF-κB, Nrf2/HO-1, signal transduction

## Abstract

Plant-derived polyphenols have attracted considerable attention because of their antioxidant, anti-inflammatory, and metabolic regulatory activities. However, despite extensive research, their biological actions are still frequently interpreted through simplified one-compound–one-pathway models, which inadequately explain their pleiotropic and context-dependent effects. Furthermore, existing studies and reviews often examine signalling pathways, structural characteristics, metabolism, and exposure biology separately, limiting a comprehensive understanding of how polyphenols regulate cellular networks. Therefore, this review aims to re-evaluate the mechanisms of representative polyphenols, including curcumin, quercetin, epigallocatechin-3-gallate (EGCG), and resveratrol, from a systems-level perspective and to examine how structural features influence signalling behaviour across different biological contexts. By integrating evidence from molecular studies, omics technologies, biotransformation research, microbiome-related metabolism, and translational investigations, we highlight that polyphenols function not as pathway-specific regulators but as context-dependent modulators of interconnected networks governing redox homeostasis, inflammation, autophagy, and energy metabolism. We further demonstrate that structural motifs such as electrophilic centres, catechol groups, gallate esters, and stilbene scaffolds influence signalling tendencies by affecting chemical reactivity, target accessibility, and metabolic fate without conferring pathway exclusivity. Based on these observations, we propose a conceptual framework of conditional signalling bias, in which polyphenol activity emerges from the dynamic interplay among chemical structure, metabolic transformation, target exposure, and network state. This framework provides a more realistic and mechanistically grounded interpretation of polyphenol action and offers a valuable foundation for future biomarker-guided, exposure-informed, and precision-oriented translational research in cardiometabolic, neurological, inflammatory, and oncological diseases.

## Introduction

Plant-derived polyphenols are a large and structurally diverse group of bioactive compounds widely distributed in fruits, vegetables, tea, coffee, cocoa, whole grains, and medicinal plants (1, 2). Owing to their antioxidant, anti-inflammatory, metabolic regulatory, and cytoprotective properties, polyphenols have attracted sustained interest in nutrition, food science, pharmacology, and disease prevention research (3–6). Epidemiological studies have consistently associated polyphenol-rich diets with reduced risks of cardiometabolic disorders, neurodegenerative diseases, cancer, and age-related conditions, further stimulating efforts to elucidate their biological mechanisms and therapeutic potential.

Over the past two decades, significant progress has been made in elucidating the molecular mechanisms underlying polyphenol action. Early studies primarily attributed their biological benefits to direct free-radical scavenging activity (7, 8). However, accumulating evidence indicates that polyphenols exert much broader effects by modulating cellular signalling pathways involved in oxidative stress responses, inflammation, autophagy, mitochondrial function, proteostasis, and energy metabolism (9). Representative compounds such as curcumin, quercetin, epigallocatechin-3-gallate (EGCG), and resveratrol have been reported to regulate signalling axes including Nrf2 (10–12), NF-κB (13–15), MAPK (16), PI3K/AKT, AMPK, mTOR, and SIRT1 (17, 18). Recent advances in transcriptomics, phosphoproteomics, metabolomics, chemoproteomics, and single-cell technologies have further revealed that these signalling responses are highly interconnected and often occur at the network level rather than through isolated pathways (19). In parallel, improvements in delivery systems, prodrug strategies, and microbiome-aware pharmacology have highlighted how stability, localization, and metabolic transformation substantially influence downstream signalling outcomes (20, 21). These developments suggest that the biological effects of polyphenols emerge from the interplay among chemical scaffold, exposure profile, cellular context, and systems-level network organization. Recent studies further support this evolving perspective. Investigations into the natural distribution of resveratrol in peanuts have highlighted the importance of dietary source and plant matrix in determining polyphenol availability and biological relevance (22). Likewise, emerging evidence indicates that polyphenols regulate ferroptosis in a context-dependent manner rather than functioning solely as antioxidants, reinforcing the concept that their biological activities are governed by cellular network state rather than individual pathways. In addition, advances in functional food materials, such as polyphenol-containing active packaging systems, further demonstrate that formulation and physicochemical properties influence polyphenol stability, exposure, and ultimately biological activity.

Despite these advances, several important challenges remain. Current interpretations frequently rely on simplified one-compound-one-pathway models, in which individual polyphenols are associated with specific signalling pathways. However, accumulating evidence suggests that such classifications are often insufficient to explain the pleiotropic and context-dependent nature of polyphenol activity. The same compound may activate distinct signalling programmes depending on dose, cell type, disease state, metabolic environment, tissue distribution, and biotransformation (23–25). Furthermore, increasing evidence indicates that microbial metabolites, conjugated derivatives, formulation-dependent exposure profiles, and host biological context substantially influence signalling outcomes (26). These observations challenge traditional pathway-centred frameworks and highlight the need for a more integrated conceptual model. Another unresolved issue concerns the relationship between chemical structure and biological activity. Structural motifs such as electrophilic centres (27), catechol groups (28), gallate esters (29), and stilbene scaffolds (30) clearly influence chemical reactivity, target accessibility, and metabolic fate (31). Nevertheless, current evidence does not support a deterministic relationship between individual structural features and specific signalling pathways. Instead, structural characteristics appear to bias signalling tendencies within dynamic cellular networks, while the final biological response is shaped by the interaction between chemical properties and biological context. Several fundamental questions remain insufficiently addressed. First, it is still unclear why the same polyphenol can produce markedly different, and sometimes even opposing, biological responses across distinct physiological and pathological settings. Compounds that exert cytoprotective effects in cardiometabolic or neurodegenerative disorders may promote oxidative stress or sensitize tumour cells under specific oncological conditions. Second, the relative contribution of parent compounds versus their metabolites remains poorly defined. Increasing evidence suggests that microbial metabolites, glucuronides, sulfates, and other biotransformation products may represent the actual bioactive species responsible for many observed signalling effects. In addition, the influence of basal metabolic state on polyphenol responsiveness remains inadequately understood. Cellular redox status, nutrient availability, mitochondrial function, inflammatory tone, and metabolic flexibility may all shape signalling outcomes, yet these factors are rarely integrated into mechanistic models. Collectively, these unresolved questions reveal a major conceptual gap in the current literature. Although increasing evidence supports the view that polyphenol activity emerges from interactions among chemical structure, metabolic transformation, target exposure, and biological context, these factors are often investigated independently rather than within an integrated framework. Consequently, the mechanisms underlying context-dependent signalling responses remain incompletely understood. Moreover, although numerous reviews have summarized the antioxidant activities, pharmacological properties, and signalling pathways associated with plant polyphenols, most continue to interpret polyphenol action from compound-centred or pathway-centred perspectives. As a result, the dynamic interactions among chemical structure, metabolic transformation, target exposure, and network-level signalling responses remain insufficiently integrated. With the increasing availability of systems-level datasets and growing recognition of context-dependent biological responses, there is a need for a more comprehensive framework capable of explaining how polyphenols influence cellular signalling across diverse physiological and pathological conditions.

Therefore, the purpose of this review is to re-examine the signalling mechanisms of representative plant polyphenols from a systems-level perspective. Rather than classifying polyphenols according to fixed pathway identities, we discuss how chemical structure, target exposure, metabolic transformation, and network state collectively determine signalling outcomes. Building upon the emerging concept that structural features bias, rather than dictate, signalling responses, we propose a framework of conditional signalling bias to explain how polyphenols function as context-dependent modulators of interconnected cellular networks. By integrating molecular, systems biology, pharmacokinetic, microbiome-related, and translational evidence, this review aims to provide a more comprehensive and mechanistically grounded framework for understanding polyphenol action and guiding future biomarker-driven and precision-oriented research.

### Literature search strategy

This review is a narrative review rather than a systematic review. Literature was identified through comprehensive searches of PubMed, Web of Science, Scopus, and Google Scholar using combinations of keywords including “polyphenols,” “curcumin,” “quercetin,” “EGCG,” “resveratrol,” “Nrf2,” “NF-κB,” “AMPK,” “mTOR,” “SIRT1,” “signalling pathways,” “multi-omics,” “metabolomics,” “microbiome,” “bioavailability,” and “biotransformation.” Priority was given to peer-reviewed articles published in English, with emphasis on recent studies and high-quality mechanistic investigations. Seminal earlier studies were also included where necessary to provide historical context or establish key biological concepts. References cited within relevant reviews and original articles were additionally screened to ensure comprehensive coverage of the literature.

### Structural determinants of conditional signalling bias

Any attempt to organize polyphenol biology by assigning individual scaffolds to discrete signalling pathways risks oversimplifying the literature. Curcumin, quercetin, epigallocatechin-3-gallate (EGCG), and resveratrol recur across studies of redox control, inflammatory signalling, metabolic sensing, autophagy, proteostasis, and cell fate regulation, with substantial overlap in the pathways implicated (3, 32, 33). A more useful question, therefore, is not if a given motif uniquely specifies a downstream signalling axis, but how chemical architecture constrains the range, probability, and context of network engagement. In this sense, structural information remains mechanistically valuable because it shapes electrophilic liability, oxidation potential, hydrogen-bonding geometry, membrane partitioning, conjugation susceptibility, and metabolic persistence, thereby conditioning target accessibility and downstream response amplitude (34). Representative examples of structurally diverse polyphenols and their associated signalling characteristics are summarized in Table 1.

**Table 1:** Structural determinants of polyphenol subclasses and their pathway-selective signalling outcomes.

Polyphenol subclass	Major dietary sources	Representative motif(s)	Primary pathway bias	Exemplar mechanistic outcome
Diarylheptanoid (Curcumin)	Turmeric	α,β-unsaturated diketone, methoxy-phenols	Nrf2/HO-1 axis	Stabilizes Nrf2 via Keap1 cysteine modification; alleviates ROS and improves cardiac, retinal, and neural injury outcomes (10)
Flavanol (Quercetin)	Onion, apple, berries	Catechol B-ring, hydroxylation pattern	Sestrin2-AMPK-SIRT1 cascade; MAPK-HO-1	Activates AMPK/SIRT1 to relieve ER stress and suppress inflammation (42); MAPK-facilitated HO-1 induction in microglia (44)
Catechin (EGCG)	Green tea	Pyrogallol + gallate ester	AKT/AMPK/mTOR; NF-κB	Remodels phosphorylation to suppress autophagy during ischemia/reperfusion; covalently blocks NF-κB p65 DNA binding
Stilbene (Resveratrol)	Grapes, peanuts, red wine	Planar stilbene backbone	AMPK-circadian coupling	Inhibits PDEs to increase cAMP-CaMKKβ-AMPK (102); promotes myotube development with circadian reprogramming (16)
Ellagitannin metabolite (Urolithin A)	Gut microbiota-derived metabolite	Microbiome-derived dibenzopyranone	AMPK-mitophagy	Induces mitophagy and engages AMPK to protect against metabolic and degenerative stress (105)
Phase II conjugates (e.g., Quercetin-3-O-glucuronide)	Generated through intestinal and hepatic glucuronidation of quercetin	Glucuronide/sulfate modifications	Context-dependent Nrf2/AMPK	β-Glucuronidase at inflamed sites regenerates active aglycone locally, enhancing tissue-specific activity (86, 106)

Curcumin exemplifies how electrophilic chemistry can bias signalling without conferring pathway exclusivity. Its *α*,*β*-unsaturated carbonyl system can react with nucleophilic cysteine residues in redox-sensitive sensor proteins (34), providing a plausible chemical basis for the recurrent association between curcumin and Nrf2/HO-1 activation in models of cardiac (10), retinal (35), and cerebral oxidative injury (36). Yet this association does not amount to pathway identity. Curcumin also modulates NF-κB-, MAPK-, and PI3K/AKT-related responses, but these intersections should not be interpreted as uniformly demonstrated direct physical interactions with all pathway components. In NF-κB signalling, curcumin has been reported to suppress IKK activity, IκBα phosphorylation and degradation, p65 nuclear translocation, and NF-κB DNA binding (37). In some systems, this may involve covalent modification of IKKβ by the electrophilic properties of curcumin or its reactive derivatives. By contrast, MAPK and PI3K/AKT involvement is more commonly inferred from altered phosphorylation of ERK1/2, JNK, p38, or AKT and downstream survival, inflammatory, or apoptotic readouts (38, 39). The immediate molecular targets upstream of these phosphorylation changes remain incompletely defined and are likely to vary with cell type, dose, redox state, and disease context. Therefore, curcumin is best interpreted as a modulator of stress-responsive circuitry through a combination of electrophile-sensitive target engagement, ROS-dependent signalling modulation, and secondary pathway crosstalk. The final biological outcome depends on the balance between adaptive signalling capacity and oxidative burden, such that curcumin may promote cytoprotection in cells with preserved antioxidant responses but induce ROS-dependent cellular stress or cytotoxicity when compensatory mechanisms are insufficient (40).

Quercetin highlights a related but distinct mode of conditional signalling bias. The catechol-rich B ring supports redox activity, metal chelation, and a protein-interaction profile compatible with engagement of stress- and energy-sensing nodes. Recurrent associations with the Sestrin2–AMPK–SIRT1 axis are well documented (17, 41, 42), but the broader literature resists reduction of quercetin action to a single dominant pathway. Quercetin has also been linked to MAPK-dependent HO-1 induction, modulation of Nrf2 activity, and suppression of NF-κB-driven inflammatory programmes (43–45), in some cases within the same biological system. Its structural contribution is therefore better understood as preferential access to nodal regulators positioned at the intersection of redox adaptation, inflammatory signalling, and metabolic stress rather than assignment to a discrete AMPK/SIRT1 pathway class.

EGCG illustrates how added structural complexity can broaden, rather than narrow, the potential modes of cellular interaction. The gallate ester increases hydrogen-bonding capacity and protein interaction potential, which may facilitate engagement with multiple molecular targets and signalling regulators. EGCG has been repeatedly associated with AKT/AMPK/mTOR remodelling, autophagy regulation, and suppression of NF-κB- and MAPK-linked inflammatory responses (15, 46–50). However, the precise relationship between the gallate moiety and these downstream signalling changes remains incompletely defined. Current evidence does not establish that the gallate ester directly determines a specific redox or stress-response outcome; rather, it appears to influence the chemical and molecular context in which EGCG interacts with cellular networks. In cerebral ischemia/reperfusion models, EGCG restrains excessive autophagy in parallel with coordinated changes in AKT/AMPK/mTOR phosphorylation (15), whereas in epithelial and macrophage inflammatory settings it attenuates transcriptional and kinase responses through partially distinct upstream mechanisms (51, 52). Thus, the gallate-bearing scaffold may increase the range of possible molecular interactions, but the resulting biological response remains determined by exposure conditions, cellular state, and pre-existing stress networks.

Resveratrol provides a complementary example in which signalling bias arises less from overt electrophilic reactivity and more from its ability to influence interconnected metabolic and stress-response pathways. Its stilbene scaffold has been associated with phosphodiesterase inhibition, cAMP/Epac/CaMKKβ signalling (53), AMPK activation (54), and circadian or transcriptional remodelling (55). Additional evidence places resveratrol within broader SIRT1- (56), mTOR- (57), and inflammation-related programmes (58), again arguing against a simple scaffold-to-pathway taxonomy. However, these associations do not indicate selective control of a single pathway. Instead, resveratrol appears to modulate interconnected energy-sensing and adaptive response networks, with the relative contribution of AMPK, SIRT1, mTOR, and related pathways varying according to cellular metabolic state, experimental conditions, and disease context. Therefore, resveratrol is better interpreted as a context-dependent regulator of metabolic and stress-response networks rather than as a pathway-specific activator.

Additional structurally diverse polyphenols further support the concept of conditional signalling bias. Apigenin, luteolin, kaempferol, fisetin, and genistein have each been reported to influence multiple signalling networks, including Nrf2, NF-κB, MAPK, PI3K/AKT, and AMPK-related pathways. However, their biological activities vary substantially across experimental systems. For example, apigenin and luteolin may exert anti-inflammatory, antioxidant, or pro-apoptotic effects depending on cellular context (59), whereas genistein can simultaneously modulate estrogen receptor signalling, metabolic regulation, and inflammatory responses (60). Likewise, fisetin and kaempferol display broad regulatory effects across oxidative stress, autophagy, and senescence-related pathways (61). These observations suggest that context-dependent network engagement is not restricted to curcumin, quercetin, EGCG, and resveratrol, but may represent a more general property of structurally diverse polyphenols. Importantly, exceptions to frequently reported pathway associations further support the need for a context-dependent framework. Although curcumin is commonly associated with Nrf2 activation, several studies have demonstrated biological effects that occur independently of Nrf2 signalling or under conditions where Nrf2 activation is minimal (62). Similarly, while resveratrol is frequently described as an AMPK activator, its biological actions may also arise through PDE inhibition (53), SIRT1-dependent regulation (63) and mTOR modulation (64) that do not require AMPK activation. EGCG likewise exhibits context-dependent behaviour, functioning as either a cytoprotective antioxidant or a pro-oxidant signalling modulator depending on concentration, cellular redox status, and disease setting (65). These apparent exceptions challenge simplistic scaffold-to-pathway classifications and suggest that recurring pathway associations should be interpreted as probabilistic tendencies rather than deterministic relationships.

Importantly, phenolic compounds are rarely consumed as isolated molecules in dietary settings but are typically present as complex mixtures within plant-derived foods. Studies examining plant-derived phenolic mixtures have demonstrated coordinated effects on oxidative stress responses (66), inflammatory pathways (67), and cellular metabolism (68), supporting the view that combinations of phenolics may generate biological outcomes that differ from those predicted by individual compounds alone. Such mixture effects further challenge reductionist interpretations of polyphenol biology, as the observed response may emerge from interactions among multiple compounds, their relative abundance, bioavailability, metabolic transformation, and the pre-existing cellular network state.

A structure-based interpretation of polyphenol activity is incomplete without considering metabolic transformation as an integral component of mechanism. Conjugation, microbial metabolism, and local deconjugation not only influence pharmacokinetics, but also determine which molecular species ultimately interacts with intracellular targets. Quercetin glucuronides, for example, may be reactivated at inflamed sites through *β*-glucuronidase-mediated deconjugation, regenerating the aglycone where stress-responsive pathways are already engaged (69). Likewise, microbiome-derived metabolites such as urolithin A display signalling properties that cannot be inferred directly from the parent ellagitannin scaffold (70). Signalling bias should therefore be regarded as an emergent property of scaffold, biotransformation, tissue microenvironment, and network state rather than as an intrinsic attribute of the nominal dietary compound alone.

The available evidence supports a restrained but mechanistically useful conclusion. Structural motifs in plant polyphenols matter because they influence electrophilicity, oxidation potential, hydrogen-bonding capacity, membrane partitioning, target accessibility, and metabolic persistence. However, these properties do not encode pathway exclusivity. Instead, they appear to alter the probability of network engagement under specific biological conditions. At present, evidence remains insufficient to establish quantitative relationships between individual structural descriptors and the likelihood of activating particular signalling networks. Whether physicochemical properties such as electrophilicity, lipophilicity, molecular flexibility, or susceptibility to biotransformation can predict signalling behaviour remains an important unresolved question. Future studies integrating chemoinformatics, chemoproteomics, systems biology, and machine-learning approaches may help establish predictive structure–network relationships and provide a more rigorous foundation for understanding context-dependent polyphenol activity. A mature structure-centred framework must therefore move beyond scaffold-to-pathway classification toward a conditional model in which recurring chemical features bias network engagement across defined biological contexts. Such a formulation is better able to accommodate pleiotropy, reconcile apparently divergent findings across experimental systems, and provide a credible conceptual basis for analogue design, delivery engineering, and biomarker-guided translation.

### Systems-level evidence for crosstalk among redox, inflammatory, and metabolic networks

**Figure 1:**
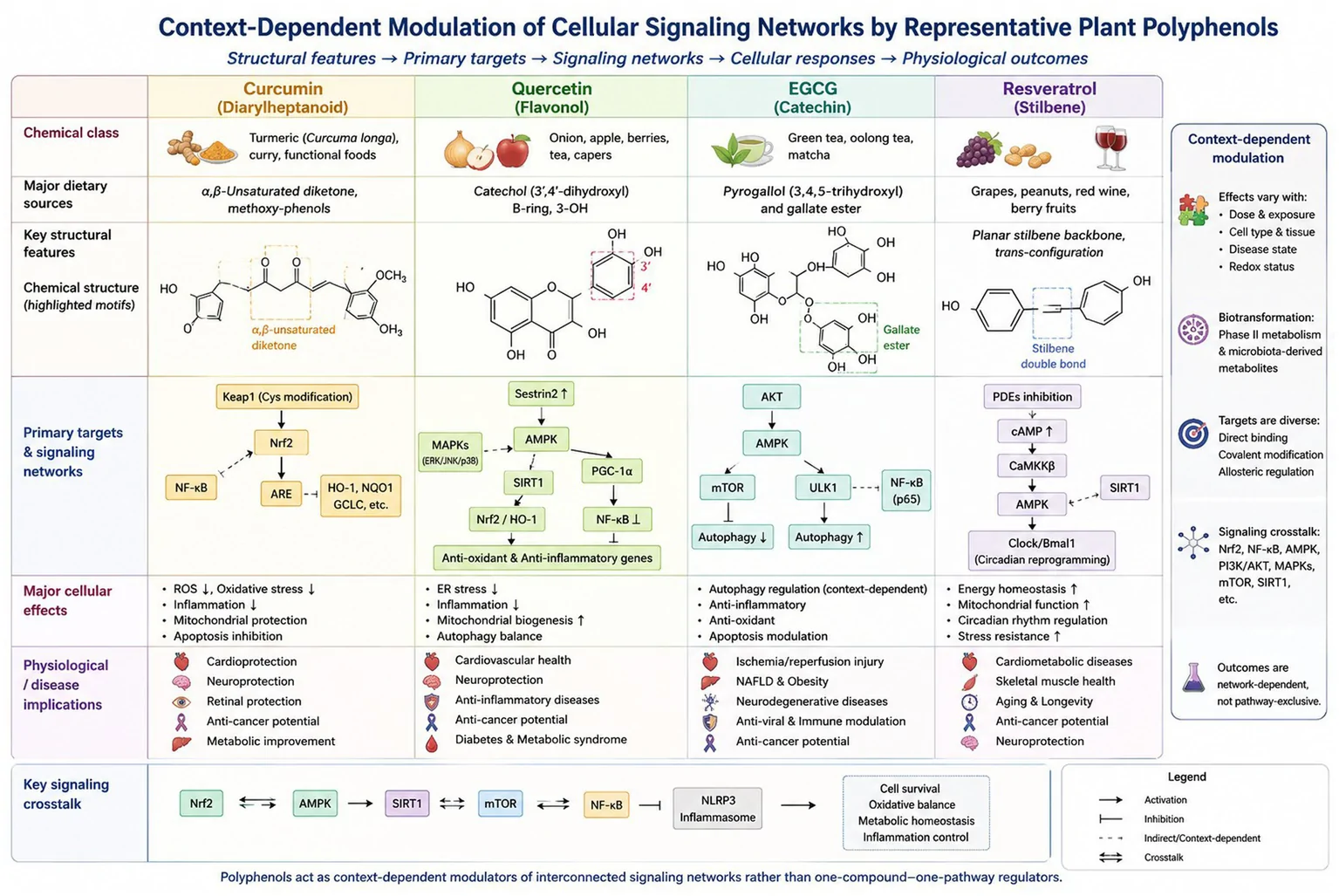
Context-dependent modulation of cellular signalling networks by representative plant polyphenols. Schematic overview of the proposed framework illustrating how representative plant polyphenols modulate interconnected cellular signalling networks in a context-dependent manner. The figure is organized from structural features-primary molecular targets-signalling networks-cellular responses-physiological outcomes. Representative polyphenols, including curcumin, quercetin, epigallocatechin-3-gallate (EGCG), and resveratrol, are shown together with their major dietary sources, characteristic structural motifs, principal molecular targets, and recurrent signalling pathways. Curcumin is primarily associated with electrophile-sensitive regulation of the Keap1-Nrf2 axis; quercetin preferentially engages the Sestrin2-AMPK-SIRT1 signalling network while modulating MAPK-dependent antioxidant responses; EGCG regulates AKT/AMPK/mTOR, autophagy, and inflammatory signalling; and resveratrol influences PDE-cAMP-CaMKKβ-AMPK, SIRT1, and circadian metabolic regulation. Despite these recurring associations, none of these compounds acts through a single pathway, and signalling responses arise from coordinated interactions among multiple regulatory nodes. The right panel summarizes the major determinants of context-dependent signalling modulation, including dose and exposure, cell and tissue type, disease state, redox environment, metabolic transformation, and microbiome-derived metabolites. Polyphenols may regulate signalling through direct target binding, covalent modification, or indirect network interactions, and the resulting biological effects depend on the pre-existing cellular network state rather than on pathway-specific activation alone. The lower panel highlights representative signalling crosstalk among Nrf2, AMPK, SIRT1, mTOR, NF-κB, and the NLRP3 inflammasome, illustrating how oxidative stress, inflammation, energy metabolism, autophagy, and mitochondrial function are integrated into a dynamic regulatory network. Collectively, the figure summarizes the central concept proposed in this review that plant polyphenols function as context-dependent modulators of interconnected signalling networks rather than as one-compound–one-pathway regulators. Solid arrows indicate activation; blunt-ended lines indicate inhibition; dashed arrows indicate indirect or context-dependent regulation; double-headed arrows denote reciprocal signalling crosstalk.

Reductionist approaches, such as single-gene or single-pathway analyses, have been essential for establishing mechanistic insights in polyphenol research. However, they are inherently limited in explaining how these compounds influence interconnected cellular systems. Polyphenols do not act through isolated pathways alone. Instead, they tend to modulate multiple signalling processes simultaneously, including redox regulation, inflammatory responses, and metabolic adaptation. The value of systems-level approaches therefore lies not in identifying entirely new pathways, but in revealing how these processes are coordinated. As illustrated in Figure 1, polyphenol activity can be viewed as an integrated network response involving interconnected modules of redox regulation, inflammatory signalling, metabolic adaptation, autophagy, and stress responses rather than isolated modulation of individual pathways. Rather than acting independently, oxidative defence, inflammation, metabolism, and stress responses are often altered together as part of an integrated network response. Although omics-based studies remain relatively limited and heterogeneous, the available evidence supports a consistent interpretation. Polyphenol activity is better understood as network-level modulation rather than the perturbation of single linear signalling cascades.

This perspective is evident in the curcumin literature. In models of diabetic cardiomyopathy (10, 71, 72) and retinal injury (35), curcumin-associated activation of Nrf2/HO-1 occurs together with attenuation of oxidative stress and inflammatory injury. These studies do not merely reinforce the importance of antioxidant signalling, rather, they suggest that redox regulation and inflammatory restraint should be interpreted as coupled outputs of the same adaptive response landscape. A similar pattern is observed with quercetin. Evidence implicating the Sestrin2–AMPK–SIRT1 axis is accompanied by reductions in cytokine production, endoplasmic reticulum stress (73), and NF-κB-linked inflammatory signalling (17, 42), indicating that metabolic sensing and immune modulation are functionally intertwined rather than mechanistically separable. Resveratrol extends these systems view by linking AMPK activation to circadian and transcriptional remodelling during myogenic differentiation (16), thereby placing polyphenol action within the temporal organization of cellular metabolism as well as within canonical stress-response pathways.

Phosphorylation-based evidence further supports a network-level interpretation of polyphenol action, although the field remains methodologically heterogeneous. In ischemia/reperfusion models, EGCG modulates AKT/AMPK/mTOR signalling in association with changes in autophagic flux (14, 15, 74), whereas phosphoproteomic studies indicate that resveratrol reshapes mTORC1-related phosphorylation patterns under nutrient stress (57). Rather than supporting pathway specificity, these findings point to a redistribution of signalling activity across kinase networks that coordinate nutrient sensing, proteostasis, and cell survival.

A systems framework also helps explain why biological outcomes vary across experimental contexts. In gastric cancer cells, depletion of Nrf2 converts curcumin from a cytoprotective modulator into a driver of ROS-dependent cytotoxicity (40). This observation illustrates that the pre-existing state of interconnected signalling networks, including basal redox balance, antioxidant capacity, inflammatory status, and metabolic conditions, can influence not only the magnitude but also the direction of polyphenol responses. Thus, the same compound may reinforce adaptive stress responses in cells with preserved defence capacity but induce maladaptive stress when compensatory mechanisms are impaired. Similarly, the anti-inflammatory effects of quercetin are enhanced by pharmacological co-activation of AMPK (17, 18, 41, 42), suggesting that metabolic background conditions the strength of downstream signalling outputs. Together, these observations support a model in which polyphenols modulate interconnected signalling networks, with functional outcomes determined by cellular context rather than by pathway-specific effects.

Host biotransformation adds a further layer of contingency. Microbiome-derived metabolites such as urolithin A engage mitophagy- and AMPK-related programmes, underscoring that the systems biology of polyphenols cannot be separated from the metabolic processing that determines which molecular species are present at the site of action. The principal contribution of omics and systems-level analysis, therefore, is not to replace mechanistic studies but to reposition them within a network framework. Polyphenols are not adequately described as isolated “Nrf2 activators” or “NF-κB inhibitors,” rather, they act as context-dependent modulators whose effects emerge from coordinated shifts across redox, inflammatory, metabolic, and proteostatic modules. Moving the field forward will require tighter integration of transcriptomic, phosphoproteomic, metabolomic, and exposure data, together with more consistent reporting of dose, timing, and bioactive species. Only under such conditions can molecular signatures become robust mechanistic biomarkers and, ultimately, support a credible basis for biomarker-guided therapeutic development.

**Figure 2:**
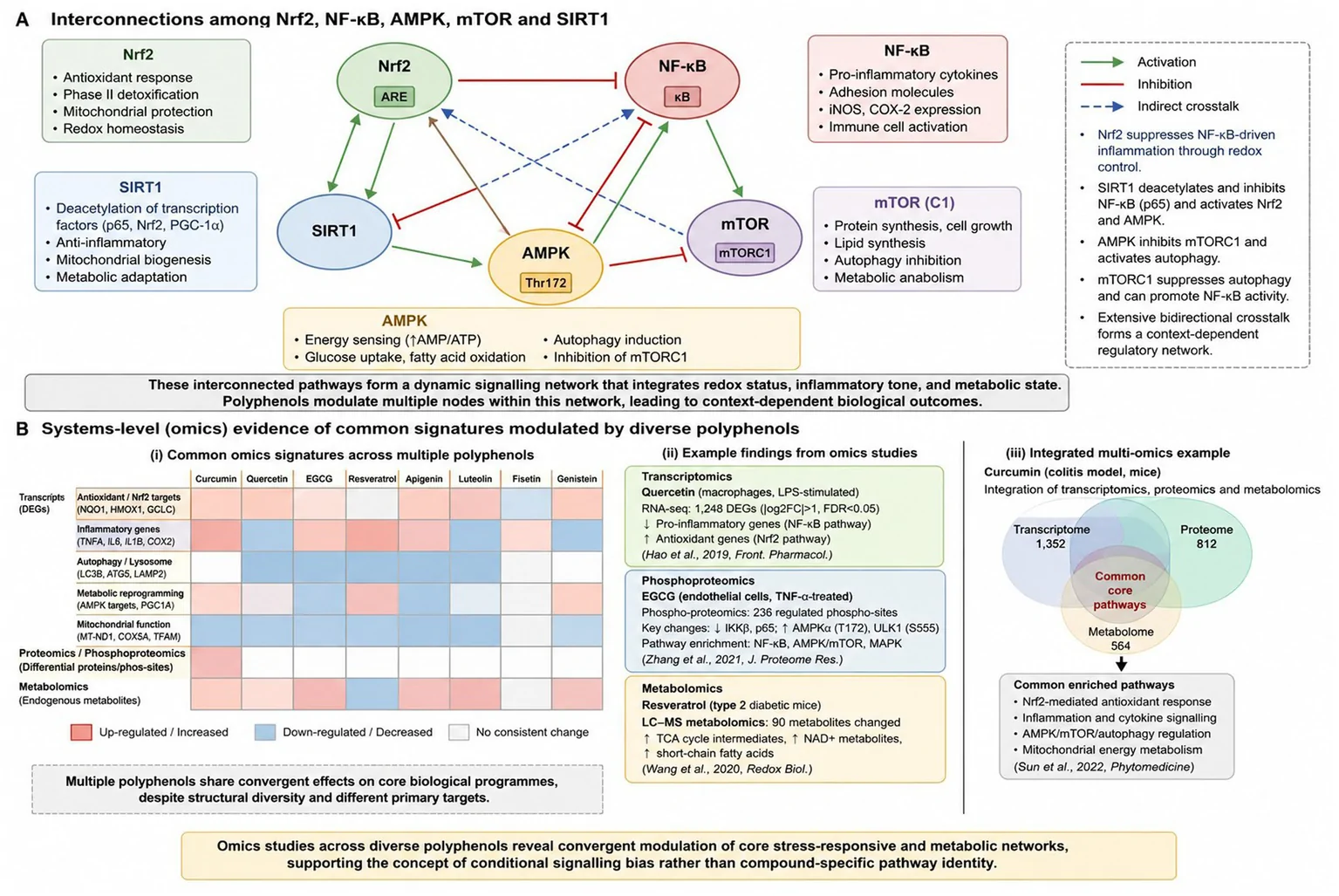
Systems-level evidence supporting context-dependent signalling modulation by structurally diverse polyphenols. **(A)** Schematic illustration of the interconnected regulatory network linking Nrf2, NF-κB, AMPK, mTOR, and SIRT1. Rather than functioning independently, these pathways exhibit extensive bidirectional crosstalk that coordinates antioxidant defence, inflammatory responses, metabolic adaptation, mitochondrial homeostasis, and autophagy. Polyphenols modulate multiple regulatory nodes within this network, leading to context dependent biological outcomes. **(B)** Summary of systems-level evidence derived from transcriptomic, phosphoproteomic, metabolomic, and integrated multi-omics studies. (i) Heatmap illustrating recurrent molecular signatures shared across representative polyphenols despite structural diversity. (ii) Representative examples of transcriptomic, phosphoproteomic, and metabolomic studies demonstrating coordinated regulation of inflammatory, metabolic, and stress-response pathways. (iii) Integrated multi-omics analyses reveal convergence of transcriptomic, proteomic, and metabolomic alterations on common biological programmes, including Nrf2-mediated antioxidant defence, NF-κB-associated inflammatory signalling, AMPK/mTOR-regulated metabolism, mitochondrial function, and autophagy, supporting the concept of conditional signalling bias proposed in this review.

Emerging omics studies further support the existence of shared systems-level signatures across structurally diverse polyphenols (75) (Figure 2). Figure 2 summarizes the systems-level framework underlying context-dependent polyphenol signalling. Panel A illustrates the extensive crosstalk among the Nrf2, NF-κB, AMPK, mTOR, and SIRT1 signalling modules, highlighting that these pathways operate as an integrated regulatory network rather than as isolated cascades. Polyphenols are proposed to influence multiple regulatory nodes within this network, thereby coordinating redox homeostasis, inflammatory responses, energy metabolism, mitochondrial function, and autophagy in a context-dependent manner. Panel B summarizes evidence from transcriptomic, phosphoproteomic, metabolomic, and integrated multi-omics studies. Across structurally diverse polyphenols, these omics platforms consistently identify convergent systems-level signatures, including activation of antioxidant defence programmes, suppression of inflammatory signalling, modulation of AMPK/mTOR-dependent metabolic regulation, remodeling of mitochondrial function, and regulation of autophagy. Although the primary molecular targets differ among individual polyphenols, integration of transcriptomic, proteomic, phosphoproteomic, and metabolomic datasets reveals common core biological pathways, supporting the concept that polyphenols function as context-dependent modulators of interconnected signalling networks rather than pathway-specific regulators. Although individual compounds differ in their primary molecular interactions, transcriptomic, phosphoproteomic, and metabolomic analyses consistently identify convergent modulation of antioxidant defence, inflammatory regulation, mitochondrial function, and autophagy-related pathways (75). Commonly enriched signatures include activation of Nrf2-associated antioxidant programmes (76), suppression of NF-κB-driven inflammatory responses (77), modulation of AMPK/mTOR signalling, and remodeling of cellular energy metabolism (78). These observations suggest that diverse polyphenols may converge on a limited set of adaptive stress-response and metabolic networks despite differences in chemical structure and immediate molecular targets.

### Delivery, biotransformation, and target exposure in polyphenol signalling

Poor aqueous solubility, chemical lability, extensive phase II conjugation, and substantial presystemic metabolism are often regarded as the principal liabilities limiting the translation of plant polyphenols (79). Yet these features are not merely pharmacokinetic obstacles. They determine which molecular species is presented to a given tissue, the temporal window over which it persists, and whether intracellular exposure is sufficient to perturb signalling networks at biologically meaningful levels. Delivery and biotransformation should therefore be considered constitutive components of mechanism rather than downstream practical constraints. In the polyphenol field, target engagement cannot be inferred from nominal scaffold identity alone; it must be interpreted through the biology of exposure.

Nanoparticle and nanoemulsion formulations provide the clearest illustration of this principle. Encapsulation strategies developed for curcumin (80), quercetin (81), and EGCG (82) have been pursued to improve solubility, protect labile phenolic functionalities, and enhance apparent biological activity in experimental systems (83). Such effects are consistent with improved preservation of chemically reactive scaffolds and altered target exposure (84). However, formulation-enhanced efficacy should not be conflated with pathway specificity. What nanocarriers more plausibly modify is the effective dose delivered to cells, the temporal window of exposure, and the chemical integrity of the species available for target engagement. In other words, delivery platforms do not assign a polyphenol to a unique signalling pathway, rather, they reshape the pharmacological conditions under which network perturbation occurs. A related principle applies to chemical masking and endogenous reactivation. Prodrug strategies are attractive in the polyphenol field because they can, in principle, stabilize reactive scaffolds and shift the site or timing of release (85). Even without formally engineered prodrugs, endogenous conjugation–deconjugation cycles already perform analogous functions *in vivo*. Quercetin glucuronides, for example, have often been interpreted as inactive clearance products, yet *β*-glucuronidase-mediated deconjugation at inflamed sites can regenerate the aglycone locally (69, 86). This observation suggests that conjugation may sometimes function less as terminal inactivation than as a transport reservoir from which bioactive species are selectively restored under permissive microenvironmental conditions. Mechanistically, the important point is that the operative signalling molecule may differ from the administered or circulating form. Beyond therapeutic nanocarriers, polyphenol-containing functional food materials and active packaging systems provide complementary examples of how formulation influences polyphenol stability and biological activity. Recent studies using pullulan-based active packaging incorporating green-synthesised nanoparticles have demonstrated that physicochemical properties, antioxidant capacity, and the stability of incorporated polyphenols are closely linked to formulation characteristics (87). Although these systems are primarily designed for food preservation rather than therapeutic delivery, they further illustrate that the biological performance of polyphenols depends not only on their intrinsic chemical structure but also on the physicochemical environment in which they are delivered. These findings reinforce the concept that exposure conditions constitute an integral component of polyphenol function rather than a secondary consideration.

Microbial metabolism introduces an additional layer that is better described as biotransformation than as delivery in the conventional pharmaceutical sense. Ellagitannins are converted by the gut microbiota into urolithin A, a more stable metabolite with signalling activities linked to mitophagy and AMPK-related programmes (70, 88). In such cases, mechanistic interpretation must follow the metabolite rather than the precursor, because the biologically relevant entity is generated downstream of host–microbe co-metabolism. This distinction is not semantic. It bears directly on how polyphenol action is conceptualized. Signalling outcomes may reflect the properties of transformed metabolites rather than those of the nominal dietary scaffold from which they originated. Receptor-associated uptake further complicates the relationship between exposure and signalling output. EGCG interaction with the 67 kDa laminin receptor provides a plausible route by which receptor abundance may modulate local sensitivity and influence downstream cGMP-linked signalling (89, 90). Even here, however, receptor engagement should not be interpreted as a deterministic route to a single cellular outcome. Rather, it represents one layer of selectivity within a broader system in which ligand stability, receptor expression, intracellular access, and pre-existing network state collectively shape the observable response.

An important unresolved issue concerns the quantitative contribution of microbiome-derived metabolism to tissue-level signalling responses. For many dietary polyphenols, circulating concentrations of the parent compounds are low because of extensive intestinal, hepatic, and microbial biotransformation (91). In contrast, microbial metabolites and phase II conjugates often achieve substantially higher systemic exposure and may represent the predominant molecular species present in tissues (92). Urolithin A, for example, is readily detected in plasma and multiple organs following consumption of ellagitannin-rich foods and has been shown to exert biological activities that differ from those of its parent compounds (93). Similarly, glucuronidated and sulfated metabolites of flavonoids frequently circulate at concentrations exceeding those of the corresponding aglycones (94). These observations suggest that signalling outcomes may be driven not only by the administered polyphenol but also by a complex mixture of host- and microbiome-derived metabolites. However, the relative contribution of parent compounds, conjugated metabolites, and microbiome-derived products to target engagement remains incompletely understood. Future studies combining tissue pharmacokinetics, stable-isotope tracing, metabolomics, and chemoproteomics will be required to identify the molecular species that directly mediate signalling responses *in vivo*.

These observations argue that exposure biology is inseparable from mechanism in the study of polyphenol signalling. Delivery systems, conjugation–deconjugation cycles, microbial metabolism, and receptor-associated uptake do not simply improve or restrict pharmacokinetics; they determine which chemical form is presented to a given cellular network, for how long, and in what microenvironmental context. A more rigorous translational framework should therefore integrate formulation properties, bioactive species, tissue pharmacokinetics, and network-level readouts within the same experimental design. Only under such conditions can altered exposure be interpreted mechanistically and linked credibly to biomarker response and disease-relevant function.

### Translational implications: disease context, biomarker dependence, and therapeutic boundaries

The translational relevance of polyphenols lies not in any claim of pathway specificity, but in the recurrent observation that these compounds perturb disease-relevant response modules at the interface of redox control, inflammatory signalling, metabolic adaptation, and proteostasis. Even so, this promise remains conditional. Most available evidence is preclinical, exposure conditions are highly model dependent, and the bioactive species present in tissues may differ substantially from the nominal parent compound because of conjugation, formulation, or microbial biotransformation. Translation should therefore be framed less as a direct extrapolation from pathway modulation to therapeutic efficacy than as a problem of linking achievable tissue exposure, biomarker response, and disease context.

#### Cardiometabolic disorders

Cardiometabolic disease provides a setting in which the signalling effects of polyphenols have been examined in relatively consistent biological contexts, particularly where oxidative stress and metabolic dysfunction coexist. In experimental models of diabetic cardiomyopathy and ferroptosis-related cardiac injury, curcumin is associated with reduced ROS burden together with activation of Nrf2/HO-1-related responses (10, 72). Quercetin and resveratrol have similarly been linked to improvements in vascular or metabolic phenotypes alongside modulation of AMPK/SIRT1-associated signalling in obesity, atherosclerotic injury, and high-fat diet models (42, 95–97). These observations suggest that polyphenol-responsive networks may be especially relevant in disease states characterized by combined redox and metabolic stress. At the same time, they highlight an important limitation. Changes in pathway readouts do not necessarily translate into functional benefit unless sufficient tissue exposure is achieved and can be linked to measurable biomarker responses. Establishing such connections between exposure, signalling, and physiological outcome remains a central requirement for translational progress.

#### Neurological injury

Acute neurological injury provides a particularly demanding context for translation, as oxidative stress, inflammatory activation, energetic failure, and dysregulated autophagy evolve over a compressed timescale (98). In models of cerebral ischemia/reperfusion, EGCG has been reported to reduce injury in association with modulation of AKT/AMPK/mTOR signalling and restraint of excessive autophagy (15). Curcumin has likewise been linked to improved outcomes after intracerebral haemorrhage, accompanied by activation of Nrf2/HO-1-related antioxidant responses (12, 99). These observations place polyphenols within a broader pattern of multi-process modulation, in which oxidative, metabolic, and inflammatory pathways are engaged in parallel rather than individually targeted. In this setting, their potential role is more consistent with adjunctive modulation of complex injury biology than with single-target neuroprotection. At the same time, the same features that make neurological injury mechanistically informative also introduce translational constraints. Blood–brain barrier penetration, therapeutic window, and formulation are likely to be decisive in determining whether these signalling effects can be translated into meaningful functional outcomes.

#### Oncology

Cancer biology illustrates the conditional nature of polyphenol efficacy. In gastric cancer, depletion of Nrf2 sensitizes cells to curcumin, leading to excessive ROS accumulation and enhanced cytotoxicity (40). This observation reframes curcumin not as a universal protector but as a biomarker-dependent probe whose activity flips with redox gene status. The translational implication is clear: patient stratification by antioxidant capacity or Nrf2 expression could predict responsiveness to curcumin. More broadly, tumours with defective antioxidant defence may be selectively vulnerable to electrophile-competent scaffolds. EGCG and quercetin add complementary axes, inhibiting NF-κB and MAPK signalling in inflammatory tumour microenvironments (100, 101), thereby reducing pro-survival transcription. Resveratrol’s PDE inhibition and AMPK activation can also antagonize tumour anabolism and proliferation (53, 102). However, the pleiotropy of these effects cautions against assuming uniform benefit. Polyphenols may protect normal tissues while simultaneously sensitizing tumours, demanding context-aware dosing and biomarker integration.

#### Metabolic and adaptive regulation

Polyphenols extend beyond stress defence to influence developmental and temporal programs. Resveratrol promotes myotube differentiation while coupling AMPK activation to circadian reprogramming (16). This mechanistic placement expands the translational horizon toward sarcopenia, metabolic syndrome, and circadian-disrupted disorders, where energy imbalance intersects with temporal misalignment. Quercetin further supports metabolic resilience by relieving endoplasmic reticulum stress and lowering inflammatory markers (103), suggesting that scaffold-specific effects can be layered to restore homeostasis at multiple levels of metabolic regulation. Urolithin A, derived from ellagitannins by the microbiome, induces mitophagy and engages AMPK in degenerative models, adding a host–microbe dimension to muscle and metabolic health. Collectively, these findings demonstrate that polyphenols can be mobilized not only as protectors against injury but also as programmers of differentiation and bioenergetics. To facilitate comparison across major disease settings, Table 2 summarizes representative polyphenols, candidate biomarkers, available clinical evidence, pharmacokinetic considerations, and long-term safety profiles relevant to their translational application.

**Table 2:** Translational relevance of representative polyphenols in disease contexts.

Disease context	Representative polyphenols	Candidate biomarkers	Clinical or human evidence	Pharmacokinetic considerations	Long-term safety profile
Cardiometabolic disorders	Curcumin, quercetin, resveratrol, EGCG	hs-CRP, IL-6, TNF-α, oxidative stress markers, lipid profile, glucose metabolism, AMPK/SIRT1-related markers	Human studies suggest potential improvements in inflammatory and metabolic biomarkers (3, 107), although effects vary across populations and formulations	Low parent-compound bioavailability; extensive glucuronidation and sulfation; formulation strongly affects exposure	Generally well tolerated at dietary or moderate supplemental doses; high-dose supplementation may cause gastrointestinal symptoms or interact with medications
Neurological and aging-related disorders	Curcumin, EGCG, resveratrol, urolithin A	Oxidative stress markers, inflammatory cytokines, mitochondrial function markers, neurotrophic factors, autophagy/mitophagy markers	Clinical evidence remains limited and heterogeneous; some human studies report changes in inflammatory or cognitive-related biomarkers (108)	Blood–brain barrier penetration and tissue exposure remain major barriers; metabolites may differ from parent compounds	Long-term efficacy and safety require further evaluation, especially for high-dose or nanoformulated preparations
Inflammatory diseases	Quercetin, curcumin, EGCG, luteolin	NF-κB-related cytokines, CRP, IL-1β, IL-6, TNF-α, immune-cell activation markers	Human biomarker studies support anti-inflammatory potential (109), but disease-specific clinical evidence is variable	Bioactive metabolites and local deconjugation may determine tissue-level activity	Usually safe at dietary levels; caution is needed for concentrated extracts and combined use with anti-inflammatory drugs
Cancer-related contexts	Curcumin, EGCG, resveratrol, genistein	Nrf2 status, ROS burden, apoptosis markers, proliferation markers, inflammatory tumor microenvironment markers	Most evidence remains preclinical (110–112); clinical studies are limited and often focus on tolerability or biomarker modulation rather than survival outcomes	Achievable tumor exposure is uncertain; formulation and tumor microenvironment may influence efficacy	Safety depends on dose, cancer type, treatment combination, and hormone-sensitive status; potential drug interactions should be considered
Metabolic flexibility and precision nutrition	Resveratrol, quercetin, urolithin A, EGCG	Metabolomic profile, microbiome-derived metabolites, insulin sensitivity, lipid metabolites, mitochondrial markers	Emerging human evidence suggests high inter-individual variability in response, partly related to metabolism and microbiome composition (113)	Microbiome-dependent metabolite production may determine responder status	Long-term personalized use requires monitoring of dose, supplement quality, medication interactions, and individual metabolic background

These disease-specific studies point to a consistent pattern. The translational relevance of polyphenols is less defined by scaffold-specific pathway assignment than by their ability to modulate disease-relevant signalling networks. Importantly, biological outcomes remain highly context dependent. Baseline antioxidant capacity, metabolic state, tissue microenvironment, microbiome composition, and the identity of the bioactive species all contribute to whether a given compound produces protective, sensitizing, or functionally neutral effects. At the same time, these findings highlight an important constraint for translation, expanding mechanistic catalogues alone is unlikely to be sufficient. Greater progress will require study designs that integrate pharmacokinetics, tissue exposure, molecular biomarkers, and functional endpoints within a unified framework. Under such conditions, polyphenols can be evaluated not as generic antioxidants or universally beneficial nutraceuticals, but as conditional modulators whose therapeutic relevance depends on biomarker-defined biological contexts.

This framework also provides a rationale for precision nutrition approaches. Rather than relying solely on the intake of parent compounds, future strategies should integrate dietary source, formulation, microbiome-dependent biotransformation, circulating metabolites, disease-specific biomarkers, and baseline metabolic status. Individual variability in metabolite generation, such as the production of microbiome-derived urolithin A or the formation of flavonoid phase II conjugates, may contribute to differences in responsiveness to polyphenol-rich diets or supplements. Therefore, precision nutrition will require biomarker-guided stratification, metabolite monitoring, and appropriate safety evaluation, particularly when concentrated polyphenol formulations or enhanced-delivery systems are considered.

### Emerging perspectives, limitations, and outstanding challenges

Despite substantial advances in polyphenol research, several important limitations continue to constrain mechanistic interpretation and translational progress. First, many experimental studies employ concentrations that greatly exceed physiologically achievable levels, raising concerns regarding the biological relevance of reported signalling effects. Consequently, the relationship between *in vitro* observations and *in vivo* target engagement remains insufficiently defined. Second, the molecular species responsible for signalling regulation are often poorly characterized. Increasing evidence suggests that microbial metabolites, phase II conjugates, and tissue-specific deconjugation products may contribute substantially to biological activity. However, most studies continue to focus on parent compounds, making it difficult to determine which bioactive species are truly responsible for observed physiological effects. Third, the complexity and context-dependency of signalling responses remain major challenges. Curcumin, quercetin, EGCG, and resveratrol frequently influence overlapping redox, inflammatory, and metabolic pathways, yet the magnitude and direction of these responses vary according to cell type, disease status, metabolic conditions, and exposure profiles. Such variability complicates mechanistic interpretation and limits the generalizability of findings across experimental system. In addition, most current studies examine individual pathways in isolation, whereas emerging evidence indicates that polyphenols regulate interconnected signalling networks. The lack of integrated analyses combining pharmacokinetics, metabolite profiling, transcriptomics, proteomics, phosphoproteomics, and functional phenotyping limits our ability to establish causal relationships between exposure and biological outcomes.

Recent studies on ferroptosis further illustrate the context-dependent nature of polyphenol activity. Natural polyphenols may suppress or promote ferroptotic responses depending on redox state, iron availability, antioxidant capacity, and disease context (104). These findings further challenge traditional antioxidant-centred classifications and suggest that polyphenol-mediated regulation of ferroptosis represents another example of conditional network modulation rather than a uniform biological effect.

## Conclusions and future perspectives

Plant polyphenols are increasingly recognized as context-dependent modulators of interconnected signalling networks rather than simple antioxidants or pathway-specific regulators. The evidence reviewed here suggests that their biological activities emerge from the dynamic interplay among chemical structure, metabolic transformation, target exposure, and network state. Accordingly, structural motifs such as electrophilic centres, catechol groups, gallate esters, and stilbene scaffolds should be viewed as determinants of signalling tendencies rather than fixed predictors of biological outcomes. This perspective provides a more comprehensive framework for understanding the pleiotropic and context-dependent nature of polyphenol action.

The concept of conditional signalling bias proposed in this review offers a useful framework for integrating seemingly divergent observations across different biological systems and disease settings. Importantly, “context” should not be regarded as an undefined or all-encompassing explanation for heterogeneous findings. Instead, we propose that it can be systematically organised into four interacting domains: chemical determinants (e.g., molecular structure and metabolic transformation), exposure-related determinants (e.g., dose, formulation, and tissue availability), cellular determinants (e.g., cell type, metabolic status, and redox balance), and network-level determinants (e.g., pre-existing signalling activity and disease state). Organising contextual variables in this manner provides clearer boundaries for interpreting experimental evidence, facilitates comparison among studies, and establishes a more structured basis for hypothesis generation and future mechanistic investigation. Rather than serving as a universal explanation for conflicting observations, this framework is intended to guide systematic evaluation of the biological factors that determine signalling outcomes.

Several important areas merit future investigation. First, future studies should move beyond describing individual signalling pathways and instead evaluate how chemical properties, exposure conditions, cellular state, and network state interact to determine biological responses under standardized experimental settings. Second, studies should prioritize identification of the bioactive metabolites and molecular species that directly mediate signalling responses *in vivo*. Third, greater emphasis should be placed on defining exposure–response relationships through integrated pharmacokinetic, pharmacodynamic, metabolomic, chemoproteomic, and spatially resolved analyses that directly link tissue exposure to network-level signalling. Fourth, the development of reliable biomarkers together with quantitative systems biology and computational modelling may enable prediction of signalling behaviour across different biological contexts, allowing the proposed framework of conditional signalling bias to evolve from a conceptual model into a predictive one. Finally, future translational studies should evaluate whether biomarker-guided stratification and precision nutrition strategies improve responsiveness to polyphenol-based interventions across diverse populations and disease settings.

Together, these efforts will facilitate a transition from descriptive catalogues of polyphenol activities toward a predictive and mechanistically grounded understanding of how dietary polyphenols regulate cellular signalling networks. By integrating structural chemistry, exposure biology, systems-level regulation, and biomarker-guided translation within a unified conceptual framework, future research may establish more robust principles for interpreting polyphenol function and accelerate the development of precision nutritional interventions and more effective therapeutic applications in cardiometabolic, neurological, inflammatory, and oncological diseases.
